# Neoadjuvant treatment in locally advanced thyroid cancer: a single institution experience

**DOI:** 10.3389/fonc.2025.1590086

**Published:** 2025-06-17

**Authors:** Ling-Feng Xu, Nuo Shi, Jia-Liang Niu, Ning An, Yi-Qing Xi, Qiu-Yi Huang, Liang Jiang, Jian Chen

**Affiliations:** Department of Head and Neck Surgery, Hubei Cancer Hospital, Tongji Medical College, Huazhong University of Science and Technology, Wuhan, China

**Keywords:** locally advanced thyroid cancer, neoadjuvant treatment, objective response rate, disease control rate, retrospective study

## Abstract

**Background:**

Locally advanced thyroid cancer is a kind of aggressive malignancy with poor overall survival (OS). Neoadjuvant therapy has shown a certain efficacy in locally advanced thyroid cancer. The objective of this study was to assess the efficacy and feasibility of surgery following neoadjuvant therapy in patients.

**Methods:**

In a retrospective study, we analyzed sixteen patients with locally advanced thyroid cancer from January 2019 to June 2024 in our institution. Among them, 7 patients with unresectable differentiated thyroid cancer (DTC) received tyrosine kinase inhibitors (TKI), and 2 patients with poorly differentiated thyroid cancer (PDTC) and 7 patients with anaplastic thyroid carcinoma (ATC) received a combination therapy of TKI, immune checkpoint inhibitors (ICI) or chemotherapy. Response and progression were evaluated by the Response Evaluation Criteria in Solid Tumors (RECIST 1.1). OS was calculated using the Kaplan-Meier method.

**Results:**

16 (4 male and 12 female) patients with locally advanced thyroid cancer were enrolled in this study, in which 10 patients (62.5%) accepted surgery following neoadjuvant therapy and 6 patients (37.5%) refused surgery. The objective response rate (ORR) was 50.00%, and disease control rate (DCR) was 81.25%. Two partial response (PR), two stable disease (SD) and one progressive disease (PD) patients achieved R0/1 resections after neoadjuvant treatment, resulting in a R0/1 resection rate of 50.00%. Grade III/IV toxicities developed in 2 of 16 patients, requiring dose reduction/discontinuation of TKI. The median OS was 17 months, with one PDTC, four ATC and six DTC patients still alive without relapse.

**Conclusions:**

Neoadjuvant treatment, including TKI, ICI or chemotherapy treatment, was safe and effective in locally advanced thyroid cancers and could create radical surgery opportunities to improve the prognosis of patients.

## Introduction

The latest statistics showed that the number of cases of thyroid cancer worldwide rose from 567,000 in 2018 to 586,000 in 2020, ranking ninth among all cancers (1, 2). According to the data, the death rate of thyroid cancer in the United States has also continued to rise since 2014 (3), and locally advanced thyroid cancer is one of the main causes of death in patients with thyroid cancer. Advanced thyroid cancer involves multiple pathological types, including anaplastic carcinoma and differentiated thyroid cancer. Differentiated thyroid cancer grows relatively slowly, shows a good response to treatment, and has a relatively favorable prognosis. Anaplastic thyroid cancer grows rapidly with high-grade malignancy, strong invasive and metastatic abilities, shows a poor response to treatment, and has an unfavorable prognosis. Locally advanced thyroid cancer often invades important surrounding structures or organs, which are difficult to be resected completely. Park et al. confirmed that patients with unresectable thyroid cancer have the shortest survival time, with a median overall survival of only 8.5 months (4). Wang et al.’s study also showed that patients who underwent R0 or R1 resection had a better 5-year survival rate than those who underwent R2 resection (94.4%, 87.6%, and 67.9%) (5). Therefore, how to create opportunities for radical surgery is the key to improving the prognosis of patients with locally advanced thyroid cancer.

Neoadjuvant treatment, including TKI, ICI or chemotherapy, is used prior to surgery in the ‘neoadjuvant’ setting. It has been confirmed that the neoadjuvant treatment has a positive outcome in lung cancer, breast cancer, rectal cancer and head and neck squamous cell carcinoma (6–10). In 2010, Cleary et al. first reported a case of neoadjuvant treatment of medullary thyroid cancer (MTC) with sunitinib, which opened the prelude to the neoadjuvant treatment in locally advanced thyroid cancer. Up to now, several studies have reported that neoadjuvant treatment for locally advanced thyroid cancer can cause tumor regression in some patients, enabling them to obtain the opportunity of surgical resection and improving their prognosis. In 2022, neoadjuvant therapy was first recommended for locally advanced papillary thyroid cancer (PTC) in the national comprehensive cancer network (NCCN) guidelines (11). The main purposes of neoadjuvant therapy for locally advanced thyroid cancer include: (1) downstaging, increasing the resectability of tumors. (2) preserving the patient’s neck organs and improving quality of life.

Currently, there are some case reports and small-scale clinical trials on neoadjuvant treatment for locally advanced thyroid cancer (12–17). Recently, a clinical trial has been reported, which evaluated the efficacy and safety of surufatinib combined with anti PD−1 antibody toripalimab in neoadjuvant treatment of locally advanced thyroid cancer (18). The results were expected to turn inoperable patients into operable, to obtain the opportunity of surgery and avoid death due to local disease progression. We present a retrospective consecutive case series of locally advanced thyroid cancer receiving neoadjuvant therapy in a real-life scenario in our institution. Within this study, almost all patients received alternative drug selection (such as anlotinib) as part of a personalized treatment strategy, with consideration of socio-economic factors and treatment availability. The study explores the feasibility of surgery and the efficacy and safety of neoadjuvant therapy.

## Methods

### Study population

The medical and pathological records were reviewed for 16 patients with locally advanced thyroid cancer from January 2019 to June 2024. Patient inclusion criteria were as follows: (i) thyroid cancer with multiple-organs invasion; (ii) R0/1 resection might not be achieved based on CT or MRI assessment; (iii) thyroid cancer with widespread lymph node metastasis; (iv) accepting neoadjuvant treatment. The exclusion criteria were as follows: (i) early resectable thyroid cancer; (ii) a history of any other cancers; (iii) not accepting neoadjuvant treatment.

### Treatment and evaluation

16 patients who received tyrosine kinase inhibitors (TKI), immune checkpoint inhibitors (ICI) or chemotherapy with a neoadjuvant proposal were included in this study. Among them, 7 patients with differentiated thyroid cancer (DTC) received TKI, 2 patients with poorly differentiated thyroid cancer (PDTC)and 7 patients with anaplastic thyroid carcinoma (ATC) received a combination therapy of TKI, ICI or chemotherapy. Thyroid cancer evaluation by MRI or CT was performed every 2–3 cycles since the first treatment. Response and progression were evaluated by the Response Evaluation Criteria in Solid Tumors (RECIST 1.1). Surgery was performed in operable patients when the surgical requirements were met. Final pathological results dictated the extent of resection: R0 (complete resection), R1 (microscopic persistence), and R2 (macroscopic residual disease). The objective response rate (ORR) was defined as percentage of patients with complete response (CR) or partial response (PR), and the DCR was defined as the proportion of patients with CR, PR or stable disease (SD). The R0/1 resection rate was defined as the proportion of patients who achieved R0 or R1. Adverse events (AEs) were recorded according to the National Cancer Institute Common Terminology Criteria for Adverse Events (NCI CTCAE) version 4.03.

### Statistical analysis

Statistical analyses were performed using SPSS version 26.0 (SPSS Inc., Chicago, IL). Survival curves were plotted using the Kaplan-Meier method. Descriptive statistics were used to summarize patients’ characteristics and AEs.

## Results

### Patients

**Figure 1:**
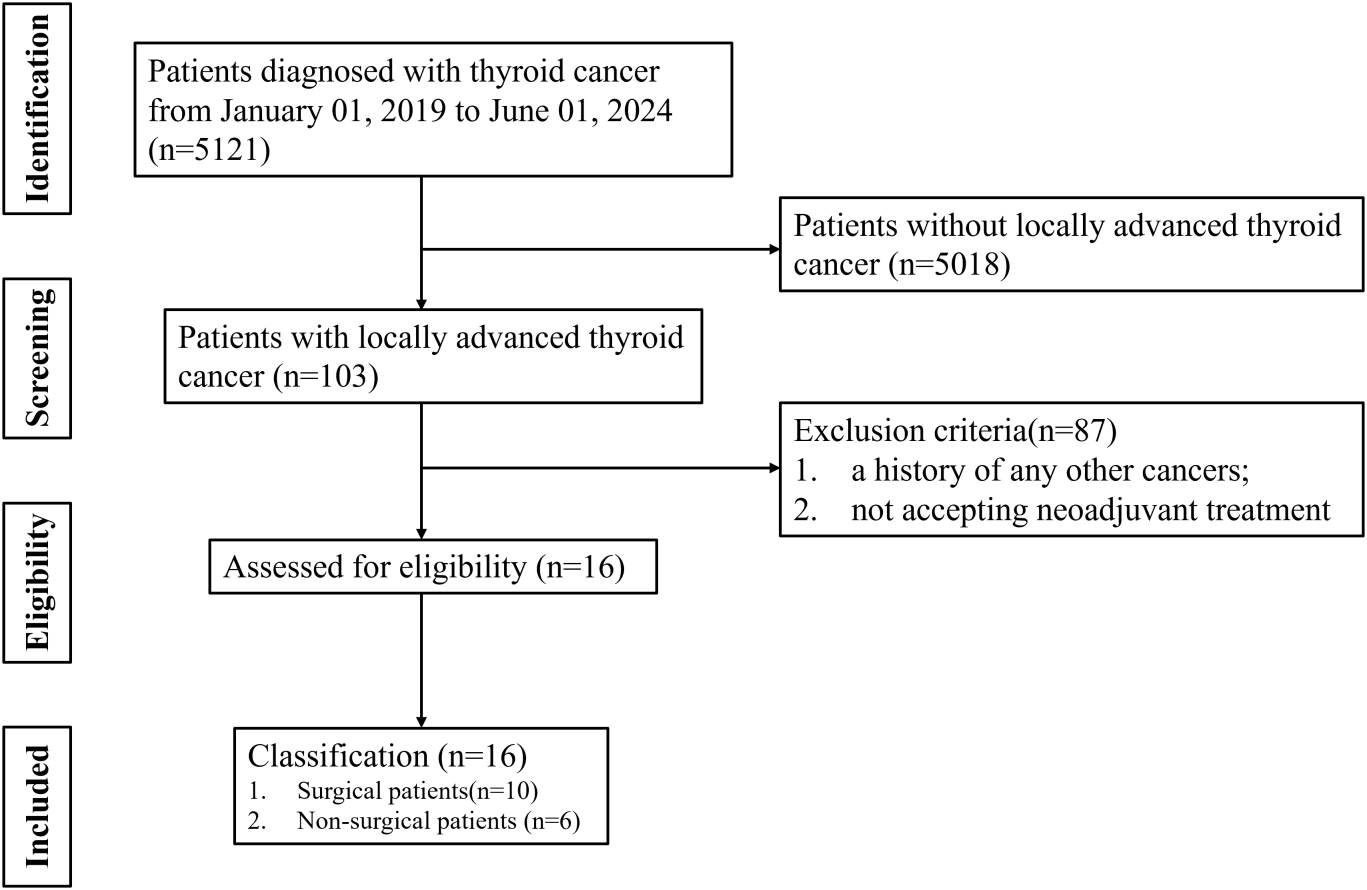
Flow diagram of case search and inclusion and exclusion criteria.

A flow diagram illustrating the case search and selection criteria are depicted in **Figure 1**. This study involved seven patients diagnosed with DTC, two patients with PDTC and seven patients with ATC. Patients were aged 35–66 years, and twelve cases were female. Nine patients (56.25%) had T4a disease and seven patients (43.75%) T4b disease. Eleven patients have undergone surgery before receiving treatment at our hospital. Patients (n=7) underwent neoadjuvant treatment with TKI, patients (n=2) received combined therapy with TKI and ICI, patients (n=1) received combined therapy with TKI and chemotherapy, patients (n=4) received combined therapy with ICI and chemotherapy, and patients (n=2) received combined therapy with TKI, ICI and chemotherapy. Baseline characteristics are listed in **Table 1**.

**Table 1:** Clinicopathological characteristics of the patients treated with neoadjuvant treatment.

Characteristics	No. of patients (n=16)
Age	
<55	4 (25%)
≥55	12 (75%)
Gender	
Male	4 (25%)
Female	12 (75%)
T stage	
T4a	9 (56%)
T4b	7 (44%)
N stage	
N1b	16 (100%)
M stage	
M0	11 (69%)
M1	5 (31%)
Structure involved	
Trachea	9 (56%)
larynx	8 (50%)
Esophagus	5 (31%)
Carotid artery	3 (19%)
Jugular vein	5 (31%)
Muscles	2 (13%)
Prevertebral fascia	1 (6%)
Previous treatment	
Surgery	11 (69%)
RAI	3 (19%)
Pathology	
DTC	7 (44%)
PDTC	2 (13%)
ATC	7 (44%)
Metastases	
Lymph node	11 (69%)
Lung	4 (25%)
Lung and bone	1 (6%)
Neoadjuvant treatment	
TKI	7 (44%)
TKI+ICI	2 (13%)
TKI+chemotherapy	1 (6%)
ICI+chemotherapy	4 (25%)
TKI+ICI+chemotherapy	2 (13%)
Post-treatment surgery	
Yes	10 (63%)
No	6 (38%)
Surgical resection	
R0	4 (40%)
R1	1 (10%)
R2	5 (50%)
Survival	
Dead	5 (32%)
Alive	11 (69%)

### Neoadjuvant treatment

The course of neoadjuvant therapy is not absolutely fixed and requires individualized decision-making based on “efficacy assessment and tolerance monitoring”. We typically use 2–4 cycles of neoadjuvant therapy, with imaging evaluation after 2 cycles to assess tumor regression and patient tolerance for surgery. Among 16 study patients, one underwent surgery after 1 cycle due to hypertension-related adverse reactions, while another delayed surgery and completed 8 cycles for personal reasons before intervention.

**Figure 2:**
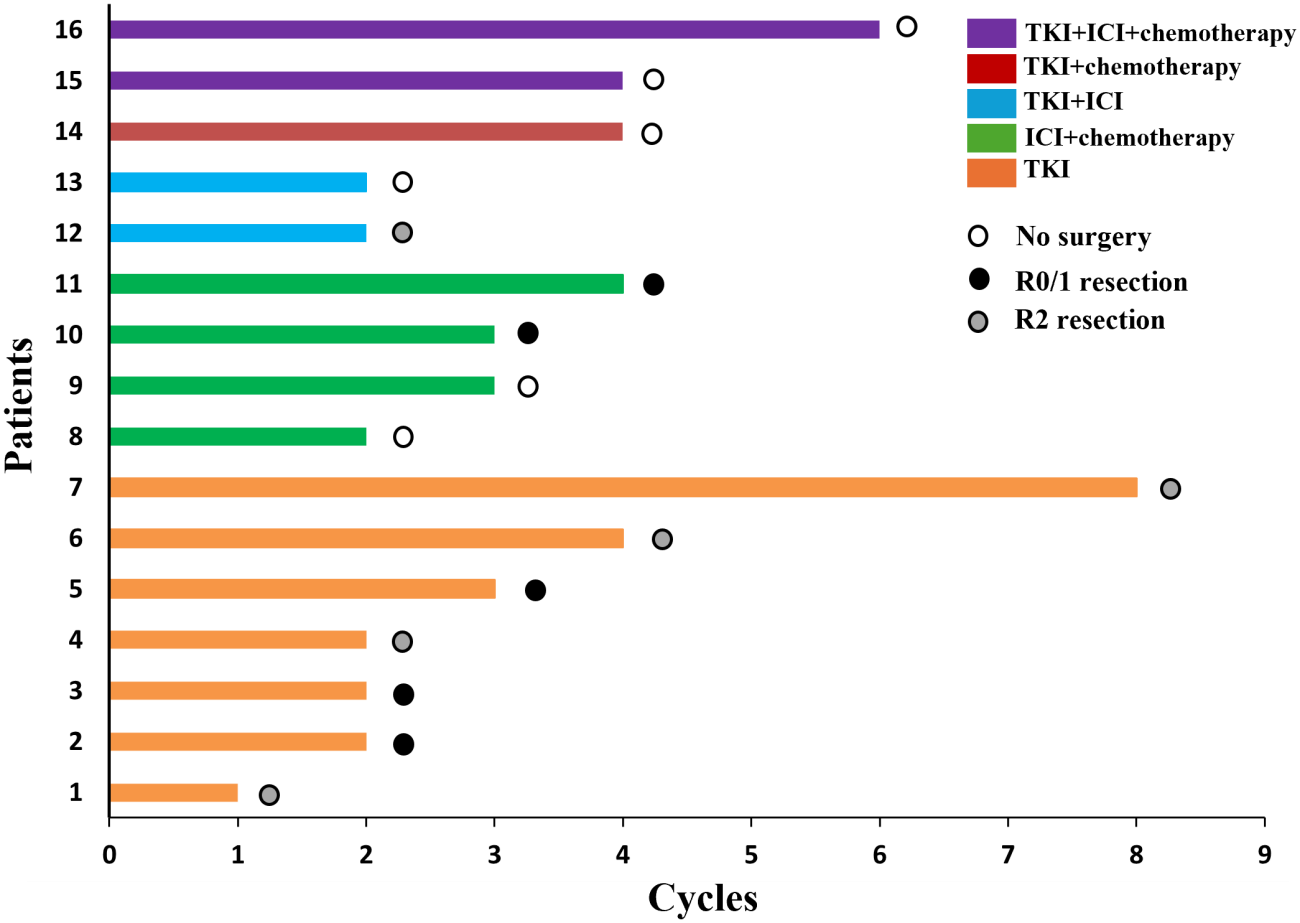
Swimmer’s plot of patients’ treatment course.

All patients received at least one (range: 1–8) cycles of TKI, ICI or chemotherapy treatment. TKI included anlotinib, savolitinib and apatinib. The chemotherapy protocol consisted of doxorubicin, cisplatin or paclitaxel. ICI is an immune checkpoint inhibitor, which targets programmed cell death protein-1 on immune cells. Toripalimab, pembrolizumab and sintilimab are available. Among them, ten patients received surgery after neoadjuvant treatment and the rest six refused to have surgery (**Figure 2**). The treatment process of the patients can be found in supplementary **Supplementary Table S1**.

### Efficacy

**Figure 3:**
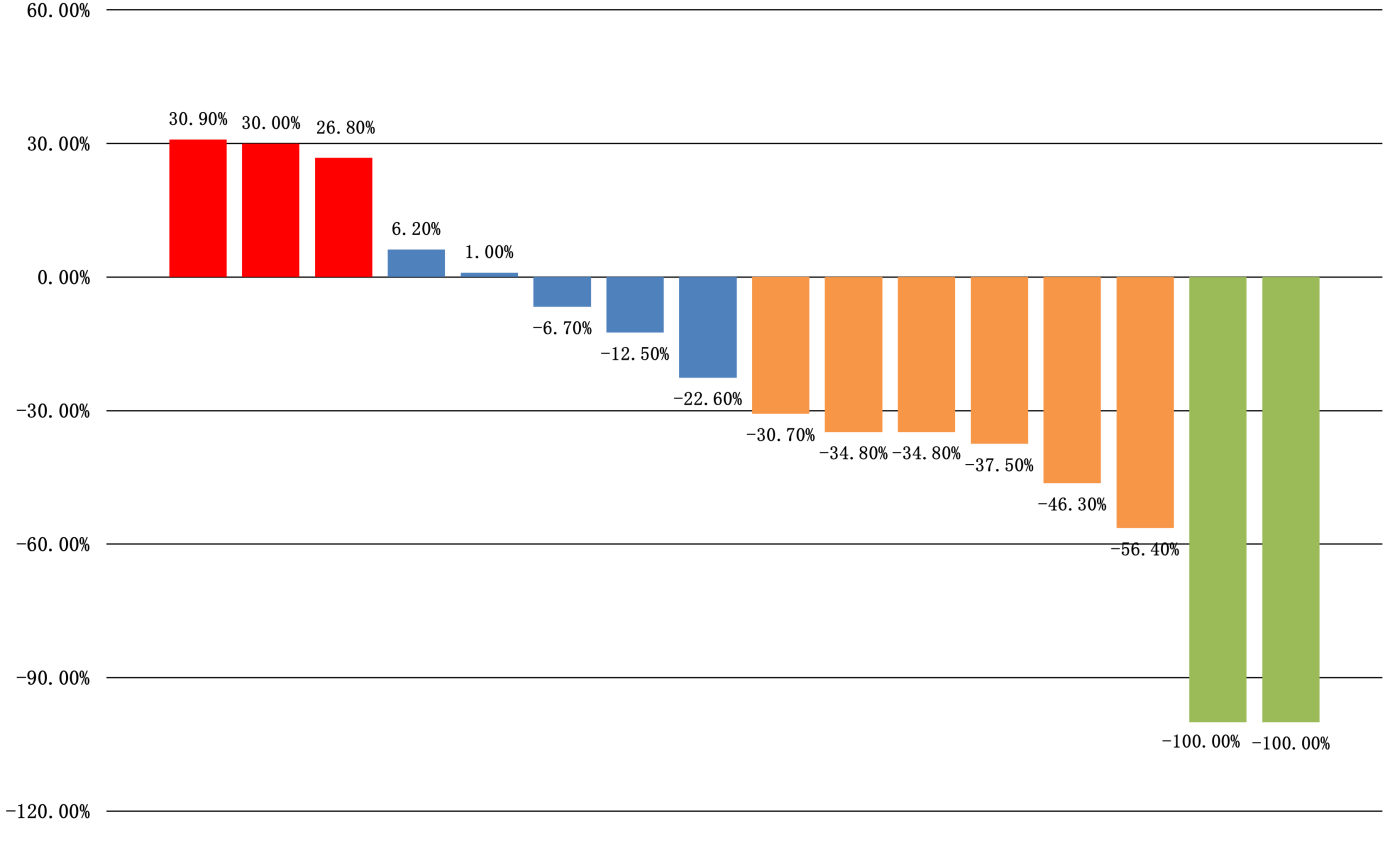
Waterfall plot of best percentage change from baseline in tumor size.

**Figure 4:**
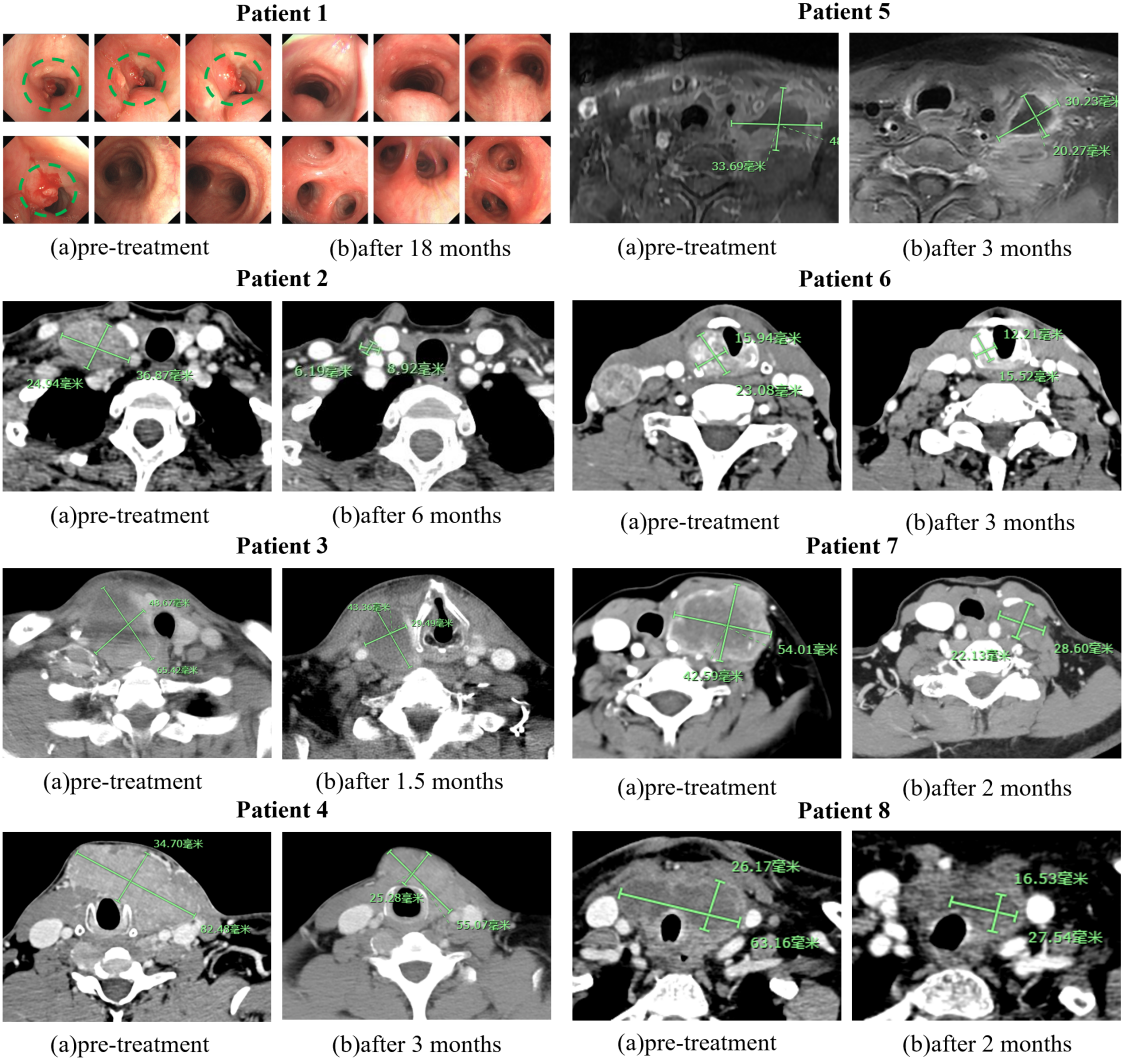
Images of 8 PR or CR before and after neoadjuvant treatment. The green markers are used to indicate the size of tumors.

**Figure 5:**
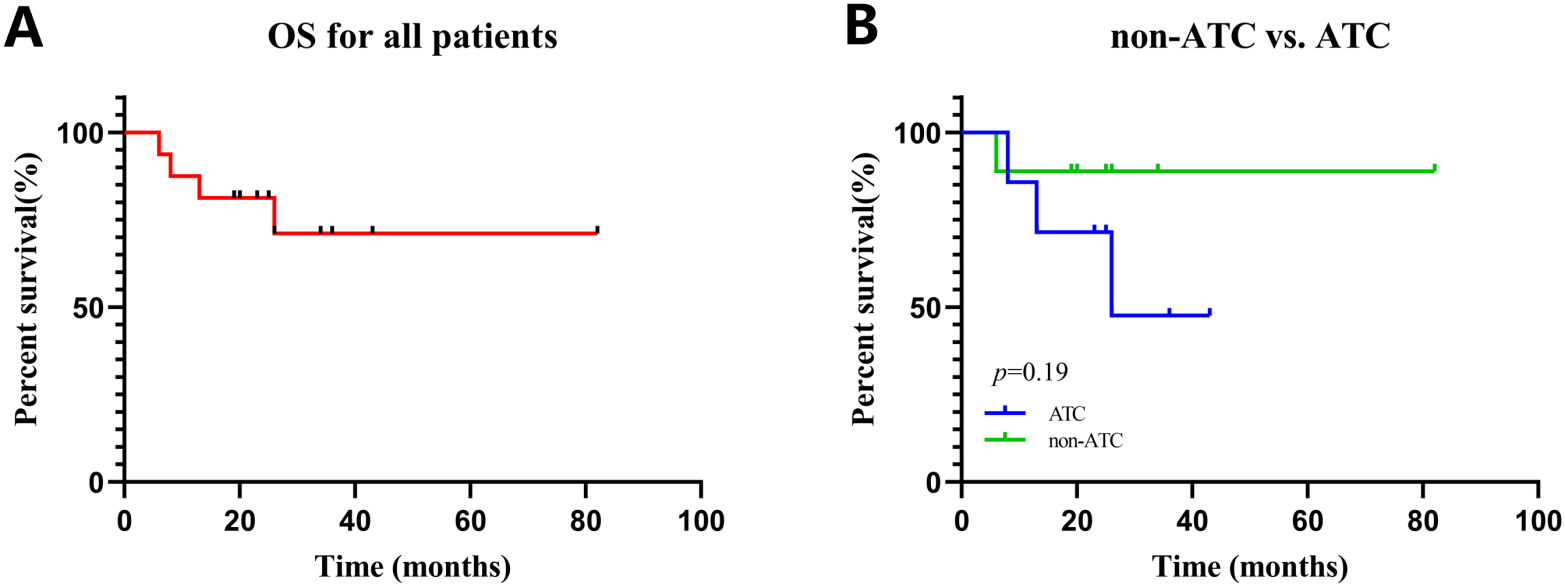
Kaplan–Meier curves for all patients. **(A)** OS in all patients. **(B)** OS in patients with non-ATC vs. ATC.

All sixteen patients were evaluable for the efficacy of neoadjuvant treatment, among whom two patients (12.50%) had confirmed CR, six patients (37.50%) PR, five patients (31.25%) SD, and three patients (18.75%) PD according to RECIST v1.1 criteria. The median best percentage change in the sum of the target lesion diameter was 41.9% in PR/CR patients (**Figure 3**). The radiographic information of the 8 patients who achieved PR or CR before and after neoadjuvant therapy is presented in **Figure 4**. The objective response rate (ORR) was 50.00%, and disease control rate (DCR) was 81.25% (**Table 2**). 6 patients (37.5%) refused surgery following neoadjuvant therapy. Two patients with CR and two patients with PR refused to have surgery because of concerns about surgical complications. One patient with PD gave up treatment. One patient with SD had a tumor enlargement of 1.0%, who refused to undergo surgery due to worrying about possible R2 resection. 10 patients (62.5%) accepted surgery following neoadjuvant therapy. Two PR, two SD and one PD patients achieved R0/1 resections after neoadjuvant treatment, resulting in a R0/1 resection rate of 50.00% (**Table 2**). The median OS for the total cohort was 25.5 (range: 6–82) months. In non-ATC group, the median OS was 26 (range: 6–82) months. In ATC group, the median OS was 25 (range: 8–43) months. The median OS for all patients who subsequently underwent surgery was 25.5 (range: 13–82) months. In non-ATC group, the median OS was 26 (range: 19–82) months. In ATC group, the median OS was 25 (range: 13–26) months. Five patients achieved R0/1 resections. The median DFS for them was 26 (range: 25–26) months. In non-ATC group, the median DFS was 26 (range: 25–26) months. In ATC group, the median DFS was 25.5 (range: 25–26) months (**Figure 5**).

**Table 2:** Best overall response of evaluable patients.

Response	No. of patients (n=16)
Best overall response	
CR	2 (12.50%)
PR	6 (37.50%)
SD	5 (31.25%)
PD	3 (18.75%)
Objective response rate (%)	50.00%
Disease control rate (%)	81.25%
R0/1 Resection Rate (%)	50.00%

### Safety

The safety profile was consistent with previously reported data. The therapy was well tolerated with the predominant grade I to IV AEs including hypertension (2/16), fatigue (3/16), oral mucositis (1/16), anorexia (3/16), joint/muscle pain (2/16), and hand–foot syndrome (1/16), alanine aminotransferase (ALT) increased (2/16), aspartate transaminase (AST) increased, total bilirubin increased (1/16), direct bilirubin increased(1/16), and proteinuria (1/16) (**Table 3**). In general, the therapy was well tolerated with the predominant grade 1 or 2 AEs, and AEs tended to resolve when neoadjuvant treatment was completed. Only two patients reported grade 4 AEs, including hypertension, fatigue, and anorexia (**Table 3**).

**Table 3:** Adverse events for all patients.

Overall adverse events	Grade I/II (%)	Grade III/IV (%)
total	16/16 (100%)	4/16 (25%)
Hypertension	2/16 (12.5%)	1/16 (6.25%)
fatigue	3/16 (18.75%)	1/16 (6.25%)
oral mucositis	1/16 (6.25%)	
anorexia	3/16 (18.75%)	2/16 (12.5%)
joint/muscle pain	2/16 (12.5%)	
hand-foot syndrome	1/16 (6.25%)	
ALT increased	2/16 (12.5%)	
AST increased	2/16 (12.5%)	
Total bilirubin increased	1/16 (6.25%)	
Indirect bilirubin increased	1/16 (6.25%)	
Proteinuria	1/16 (6.25%)	

Dose interruption and reduction due to AEs occurred in two patients. One 58-year-old female patient had grade 4 hypertension and anorexia but was resolved after dose reduction of anlotinib. Another 59-year-old female patient had dose interruption due to AEs of fatigue and anorexia. No patient experienced AE leading to fatal AE.

### Surgery

Among ten patients who had surgery after neoadjuvant treatment, thyroidectomy, and central/lateral neck dissection were performed in seven patients. Another three patients who had previous total thyroidectomy received reoperation of central/lateral compartment dissection. Tracheal resection was performed in two patients and end-to-end anastomosis was then performed to repair the trachea. One patient sacrificed one side of the internal jugular vein, one had tracheotomy, two had partial laryngectomy, and two had upper mediastinal lymph node dissection. In two patients who underwent partial laryngectomy, the effect of neoadjuvant therapy was not satisfactory. One of them achieved PD, and the other achieved SD. The recurrent laryngeal nerves were preserved in all ten patients. Among them, five patients could only achieve an R2 resection because they needed to preserve the recurrent laryngeal nerve. No patient received a blood transfusion. The median postoperative hospitalization time was 15.5 (range: 8–28) days, and five patients were transferred to intensive care unit (ICU).

## Discussion

For patients suffering from locally advanced thyroid cancer, R0 or R1 resection can provide a longer survival period and achieve the best palliative effect. However, most patients with locally advanced thyroid cancer have already missed the opportunity for radical surgery. Therefore, creating the opportunity for radical surgery remains a crucial point in the treatment. Neoadjuvant therapy is a novel approach for locally advanced tumors, which can reduce the tumor volume, lower the stage, increase the R0/R1 resection rate, and preserve the functions of vital organs at the same time. Moreover, radical thyroidectomy following neoadjuvant therapy renders radioactive iodine therapy or radiotherapy feasible for locally advanced or metastatic thyroid cancer. Based on our single-center experience, neoadjuvant therapy could cause tumor regression in a large proportion of patients, manifested as a reduction in tumor volume. Among the patients who underwent surgery after neoadjuvant treatment, we found that tumor regression reduced the difficulty of surgery, which made R0/1 resection possible. In our observations, the overall ORR and DCR were 50.00% and 81.25% respectively, and the R0/1 resection rate reached 50.00%, which were satisfactory and consistent with previous research findings (18, 19).

In this study, the median OS of ATC and non-ATC patients is almost the same. The reasons are as follows. The median OS is a commonly used indicator for evaluating the survival status of tumor patients. However, it has some limitations. When the sample size is small, the estimated value of median OS may be unstable and is likely to be affected by individual extreme values or the survival conditions of a small number of patients, resulting in a large deviation in the results. Two ATC patients who achieved CR were diagnosed in 2021 and 2022 respectively, and their OS reached 43 months and 36 months respectively. Most of the patients in the subgroup of non-ATC patients were diagnosed in 2023. Therefore, the follow-up time for the subgroup of non-ATC patients is relatively short. Besides, the sample size is small. There are only 9 non-ATC patients and 7 ATC patients.

For patients who underwent surgery and those who did not, there was no difference in terms of DFS and OS. The lack of a significant difference in survival may also be due to insufficient follow-up time or insufficient statistical power. If the follow-up period is too short, it may not be possible to observe the long-term effects of surgery and neoadjuvant treatment on survival. In addition, if the sample size is small, the study may not have enough statistical power to detect survival differences between the two groups. The survival of cancer patients is influenced by multiple factors other than surgery and neoadjuvant treatment. These factors include the patients’ overall health status, the effectiveness of any subsequent adjuvant treatment or palliative care. If these other factors are not properly considered or balanced between the two groups, they may confound the results, giving the appearance that there is no survival difference related to the surgery.

In the neoadjuvant treatment of thyroid cancer, a variety of agents and their combinations have been studied. Tyrosine Kinase Inhibitor (TKI) was the first kind of small molecule targeted drugs being reported, and there have been reports on the application of TKI in different pathological subtypes of thyroid cancer. In 2017, Tsuboi et al. first reported a case of locally advanced papillary thyroid carcinoma treated with neoadjuvant therapy using lenvatinib. Prior to the treatment, the extent of tumor invasion into the cervical esophagus and trachea of this patient was evaluated. After 18 weeks of neoadjuvant therapy, the primary tumor and cervical lymph nodes shrank by 84.3% and 56.0% respectively (13). Similarly, Iwasaki et al. and Stewart et al. also reported cases of papillary thyroid cancer treated with neoadjuvant therapy using lenvatinib. In both cases, complete tumor resection was achieved after the administration of the drug (20, 21). In 2019, it was reported that six patients with undifferentiated cancer carrying BRAF gene mutations achieved complete surgical resection after treatment with the dual-targeted drugs dabrafenib and trametinib. Subsequently, they received postoperative adjuvant radiotherapy and chemotherapy. The overall survival rates at 6 months and 1 year were 100% and 83% respectively, and the local control rate reached 100% (22). Golingan et al. reported a case of neoadjuvant therapy with lenvatinib for medullary thyroid carcinoma. The patient had a large mass in the left thyroid, along with metastasis to the central and left lateral lymph nodes. After 4 months of neoadjuvant therapy, the tumor volume decreased by nearly 70%. Subsequently, the tumor was completely resected through surgery. During the 4-month follow-up after surgery, the calcitonin level decreased by 99%, and no residual lesions were detected by imaging during the 6-month follow-up (23). In our retrospective study, seven patients with unresectable differentiated thyroid cancer (DTC) received only neoadjuvant targeted therapy. According to the RECIST v1.1, one patient achieved a partial response (PR), four patients had stable disease (SD), and two patients had progressive disease (PD).

Recently, a new approach in neoadjuvant treatment has emerged. It involves the combination of TKI, ICI or chemotherapy. For instance, lenvatinib and pembrolizumab have been used to treat ATC and PDTC, yielding favorable results (19). It’s reported that PD-L1 is generally expressed in pT4-stage thyroid cancer. PD-L1 positivity rate was higher in ATC than in PTC within the same studies, which indicated that ICI was a novel immunotherapy target for optimizing the management landscape of ATC (24). Compared with single-agent therapy, neoadjuvant immunotherapy combination demonstrated remarkable efficacy in our treatment cohort. In our retrospective study, nine patients with ATC or PDTC received neoadjuvant immunotherapy combination. According to the RECIST v1.1, two patients achieved CR, five patients achieved PR, one patient had SD, and one patient had PD. The remission rates observed in neoadjuvant immunotherapy combination are similar to the previously published data of other pretreated solid tumors, such as metastatic renal cell carcinoma and hepatocellular carcinoma (25, 26). In our study and previously published ones, CR and long-lasting remissions were rarely observed in patients treated with TKI alone (27–29). The addition of ICI most likely contributed to this effect and increased the response rate.

Overall, the combination treatment was well tolerated. In our retrospective study, the most frequently reported treatment-related adverse events were fatigue and anorexia. Grade III/IV AEs that led to dose interruption and reduction were reported in two patients. No patient experienced an AE that led to treatment discontinuation or a fatal AE. Neoadjuvant therapy did not significantly increase the incidence of AEs. Preoperative medication did not increase the difficulty of surgery or the risk of postoperative complications such as bleeding. In the neoadjuvant treatment of locally advanced thyroid cancer, the combination of TKI, ICI or chemotherapy was well-tolerated with manageable toxicities.

Although our study demonstrated that neoadjuvant therapy is safe and effective in locally advanced thyroid cancer and can create opportunities for radical surgery to improve the prognosis of patients, there are still some limitations in this study. Firstly, the nature of retrospective study may introduce selection bias. Additionally, the study lacks data on tumor mutational burden (TMB) and the expression level of PD-L1, both of which are independent biomarkers for the response to immune checkpoint inhibitors in solid tumors.

## Conclusions

In this case series, nearly all patients had a good tolerance to the combination therapy of TKI, ICI or chemotherapy. It should be noted that the use of alternative TKIs selection (e.g., anlotinib, savolitinib and apatinib), influenced by socio-economic determinants, highlights the need for flexible treatment algorithms in resource-limited settings. The results are encouraging, with an ORR of 50%, a DCR of 81.25%, and an R0/R1 resection rate of 50.00%. Based on the promising results, our future plan is to expand the sample size and conduct multicenter clinical research. We hope to further evaluate the efficacy and feasibility of neoadjuvant therapy in locally advanced thyroid cancer and whether it can become a standard treatment protocol.

## Data Availability

The raw data supporting the conclusions of this article will be made available by the authors, without undue reservation.
