# Supplementary material for: Neoadjuvant treatment in locally advanced thyroid cancer: a single institution experience
Source: Front Oncol. 2025 Jun 17;15:1590086. doi: 10.3389/fonc.2025.1590086 (PMC12209174; doi:10.3389/fonc.2025.1590086)
Supplement: Supplementary file 1 [file Table1.docx]

**Supporting information**

**For**

**Neoadjuvant Treatment in Locally Advanced Thyroid Cancer: A Single Institution Experience**

| **Patients** | **Pathology** | **Prior treatment** | **Neoadjuvant treatment** | **Therapeutic cycles** | **BOR** | **TNM** | **R0/1/2 resection** | **Outcome** |
| --- | --- | --- | --- | --- | --- | --- | --- | --- |
|  |  |  |  |  |  |  |  |  |
| 1 | DTC | Surgery+RAI | TKI | 1 | PD | T4aN1M0 | R2 | Alive |
| 2 | DTC | Surgery+RAI | TKI | 2 | PD | T4aN1M1 | R0 | Alive |
| 3 | DTC | Surgery | TKI | 2 | SD | T4aN1M1 | R0 | Alive |
| 4 | DTC | — | TKI | 2 | SD | T4aN1M1 | R2 | Alive |
| 5 | DTC | Surgery | TKI | 3 | SD | T4aN1M0 | R0 | Alive |
| 6 | DTC | Surgery+RAI | TKI | 4 | PR | T4bN1M1 | R2 | Alive |
| 7 | DTC | — | TKI | 8 | SD | T4aN1M1 | R2 | Alive |
| 8 | PDTC | — | ICI+chemotherapy | 2 | PD | T4aN1M0 | — | Dead |
| 9 | ATC | Surgery | ICI+chemotherapy | 3 | PR | T4aN1M0 | — | Alive |
| 10 | ATC | — | ICI+chemotherapy | 3 | PR | T4aN1M0 | R0 | Dead |
| 11 | ATC | Surgery | ICI+chemotherapy | 4 | PR | T4bN1M0 | R1 | Alive |
| 12 | ATC | — | TKI+ICI | 2 | PR | T4bN1M0 | R2 | Dead |
| 13 | ATC | Surgery | TKI+ICI | 2 | CR | T4aN1M0 | — | Alive |
| 14 | ATC | Surgery | TKI+chemotherapy | 4 | SD | T4bN1M0 | — | Dead |
| 15 | PDTC | Surgery | TKI+ICI+chemotherapy | 4 | PR | T4aN1M0 | — | Alive |
| 16 | ATC | Surgery | TKI+ICI+chemotherapy | 6 | CR | T4aN1M0 | — | Alive |

**Table S1**. The therapeutic course of the patients.
